# The effect of sesame oil consumption compared to sunflower oil on lipid profile, blood pressure, and anthropometric indices in women with non-alcoholic fatty liver disease: a randomized double-blind controlled trial

**DOI:** 10.1186/s13063-022-06451-1

**Published:** 2022-07-08

**Authors:** Hamid Vahedi, Masoumeh Atefi, Mohammad Hassan Entezari, Akbar Hassanzadeh

**Affiliations:** 1 Department of Gastroenterology, School of Medicine, Shahroud University of Medical Sciences, Shahroud, Islamic Republic of Iran; 2 Department of Clinical Nutrition, School of Nutrition & Food Science, Isfahan University of Medical Sciences, Isfahan, Islamic Republic of Iran; 3 Food Security Research Centre and Department of Clinical Nutrition, School of Nutrition & Food Science, Isfahan University of Medical Sciences, Isfahan, Islamic Republic of Iran; 4 Department of Epidemiology and Biostatistics, School of Health, Isfahan University of Medical Sciences, Isfahan, Islamic Republic of Iran

**Keywords:** Sesame oil, Lipid profile, Blood pressure, Anthropometric indices, Non-alcoholic fatty liver disease

## Abstract

**Background:**

Non-alcoholic fatty liver disease (NAFLD) is one of the most common liver diseases in the world. There is strong evidence that dyslipidemia and other cardio-metabolic disorders are highly prevalent in patients with NAFLD. This trial aimed at examining the effect of sesame oil (SO) in the context of a weight loss program on lipid profile, blood pressure, and anthropometric indices in women with NAFLD.

**Methods:**

This randomized, double-blind, controlled trial was carried out on 60 women with NAFLD. Subjects were randomly assigned to the SO group (*n* = 30) and sunflower oil (SFO) group (*n* = 30), each person consuming 30 g of oil per day for 12 weeks. All the participants received a hypocaloric diet (− 500 kcal/day) during the study. Lipid profile, blood pressure, and anthropometric indices were assessed at pre- and post-intervention phases.

**Results:**

In total, 53 participants completed the study. Following 12 weeks of intervention, anthropometric indices (*p* < 0.001) and systolic blood pressure (SBP) (*p* < 0.05) were significantly decreased in both groups and diastolic blood pressure (DBP) was significantly decreased in So group (*p* = 0.03). There was no significant change in lipid profile in both groups (*p* > 0.05). After adjusting for confounders, DBP (*p* = 0.031) and total cholesterol (TC) divided by high-density lipoprotein cholesterol (HDL-C) (*p* = 0.039) in the SO group were significantly reduced compared to the SFO group (*p* < 0.05).

**Conclusions:**

The present clinical trial revealed that SO and SFO may not differently affect anthropometric indices, SBP, and lipid profile except for TC/HDL-C. In addition, SO may be effective in improvement of DBP and TC/HDL-C compared to the SFO group.

**Trial registration:**

Ethical approval of this trial was obtained at Isfahan University of Medical Sciences with the reference number of IR.MUI.RESEARCH.REC.1399.548 (https://ethics.research.ac.ir/ProposalCertificateEn.php?id=158942&Print=true&NoPrintHeader=true&NoPrintFooter=true&NoPrintPageBorder=true&LetterPrint=true), and it was registered before the start of the patient recruitment on December 12^th^, 2020 in the Iranian Registry of Clinical Trials (IRCT) with the registration number of IRCT20140208016529N6.

## Introduction

NAFLD is known to be the leading cause of liver-related deaths and is one of the most common liver diseases in the world [1–4]. The prevalence of this disease throughout the world and Iran, which is the host of this clinical trial, is 25% and 33.9%, respectively [5, 6]. There are strong evidences that cardio-metabolic disorders are highly prevalent in patients with NAFLD [7]. Among patients with NAFLD, the prevalence of obesity, hypertension, and hyperlipidemia is 67, 46.7, and 77.1%, respectively [8]. These comorbidities have extensive negative effects on NAFLD. There are common pathogenic mechanisms and similar biological processes between NAFLD and these markers [5]. In fact, the severity of NAFLD is strongly influenced by obesity, which leads to the worsening of liver fibrosis and the progression of liver diseases. The presence of NAFLD is also effective in the development of cardiovascular diseases (CVD) [5]. Studies have shown that individuals with NAFLD had a higher risk of heart disease than individuals without NAFLD [9].

The results of pharmacological studies for NAFLD treatment have remained inconclusive [10]. To date, the best proven treatment for the management of NAFLD is lifestyle modification (having physical activity and a healthy diet) [1, 10, 11]. Among macronutrients, fats have a great impact on chronic diseases such as CVD and NAFLD [12]. Types and amount of the dietary fats play an important role in fat accumulation in the liver, which are responsible for 15% of the total liver fat content [13]. High fat diet is well known as a dietary factor in the progression of hepatic steatosis [14]. Changes in dietary fatty acids may play an important role in the prevention and treatment of coronary heart diseases, hypertension, and fatty liver disease [10, 15, 16]. Monounsaturated fatty acids (MUFAs) and polyunsaturated fatty acids (PUFAs) are parts of a complete diet that have beneficial effects in preventing the occurrence and progression of NAFLD [10].

SO is used as a healthy oil in Asian countries [17] and is a good source of MUFAs and PUFAs (83–90% of fatty acids), which contains 36–54% oleic acid and 38–49% linoleic acid. PUFAs of SO improve the lipid profile by increasing HDL-C and decreasing LDL-C, TG, and fat oxidation [18]. MUFAs reduce TG by increasing the oxidation of fatty acids [19]. SO is rich in natural antioxidants such as tocopherol, polyphenols, phytosterols, flavonoids, and lignans [20] and minerals such as magnesium, calcium, copper, iron, and zinc [11], which reduces blood pressure, hyperlipidemia, and lipid peroxidation by increasing enzymatic and non-enzymatic antioxidants [11]. Sesamin in SO has anti-atherosclerotic properties [21] and may control blood pressure [22, 23].

Animal studies have shown that SO lignans protect the liver by the decrease in lipogenesis and lowering the concentration of cholesterol in the liver [14, 24]. A number of studies have shown that SO improves TC, LDL-C [22, 25, 26], TG [23, 25, 26], HDL-C [25–27], and blood pressure [20, 28]. However, some studies were unable to confirm this effect on TC and LDL-C [28, 29], TG [22, 27, 29] HDL-C, [20, 28, 29], and blood pressure [29]. A number of animal studies have examined the beneficial effects of SO on liver diseases [11, 30–32]. To the best of our knowledge, no human study has examined the effect of SO on cardio-metabolic biomarkers in patients with NAFLD. Therefore, the present clinical trial investigates the effect of SO on cardio-metabolic biomarkers including lipid profile, blood pressure, and anthropometric indices in women with NAFLD.

## Materials and methods

### Study design and patients’ characteristics

The study was a randomized, double-blind, parallel, controlled trial in Shahroud, Iran. The current study aimed to assess the effects of SO on lipid profile, blood pressure, and anthropometric indices in women with NAFLD. SPIRIT (Standard Protocol Items: Recommendations for Interventional Trials) was used as a framework for reporting the present protocol [33]. The ethical approval of this trial was obtained from the ethics committee of Isfahan University of Medical Sciences on November 9^th^_,_ 2020, with a reference number of IR.MUI.RESEARCH.REC.1399.548 and was registered in the Iranian registry of clinical trials (https://www.irct.ir/trial/52288) with the registration code of IRCT20140208016529N6 on December 12^th^, 2020.

Sixty women with NAFLD were recruited from the liver and gastrointestinal clinics of Shahroud, Iran. The mean of age was 39 years, and the mean of BMI was 31.3 kg/m^2^.

Female participants, being 20 to 50 years old, having 1–3 fatty liver grade by examining ultrasonography, consuming SFO as the routine oil, having BMI between 25 and 40, were included in the study (inclusion criteria).

The exclusion criteria applied to the potential participants were as follows: having been smokers, alcohol consuming, menopausal, pregnant, breastfeeding; having undergone insulin therapy throughout the study period; having hormone-dependent cysts and allergies, history of breast cancer, sclerosing cholangitis, renal failure, autoimmunity, malignancies, celiac disease, hereditary hemochromatosis (transferrin saturation greater than 45%), Wilson’s disease, liver diseases (cirrhosis, alcoholic liver disease, viral hepatitis, hepatitis, primary biliary cirrhosis, biliary obstruction and liver damage induced by hereditary hemochromatosis drugs); have consumed hepatotoxicity drugs such as tamoxifen and lithium, drugs affecting the levels of liver enzymes ALP, AST, and ALT including valproic acid, 3-hydroxy-3-methylglutaryl coenzyme A reductase inhibitors, acetaminophen, salicylates, phenytoin, benzodiazepines, drugs causing fatty liver such as methotrexate, tamoxifen, valproate, drugs such as corticosteroids, amiodarone, perhexiline, aspirin, hydralazine, contraceptives, estrogen; have participated in other studies in the last 6 months; having any weight loss diet or special diet in the last 3 months; and consuming multivitamin mineral and omega-3 supplements 3 months prior the trial.

The participants who lost to follow-up the study for any reason or had improper adherence were excluded from the analysis (drop-out criteria).

At the first visit, all study protocols were explained to the participants, and written consents, demographic information, and their medical histories were obtained. During the run-in, participants used routine oil (SFO) and healthy eating recommendations. SFO is widely produced and distributed in Iran and is well known as the main type of oil consumed by Iranians [34].

### Chemical analysis of SO and SFO

Fatty acids composition of SO and SFO (Kamjed Company, Shahroud, Iran) were evaluated using high-performance gas chromatography at the reference food chemistry laboratory (ViroMed Specialized Laboratories, Tehran, Iran). The percentages of PUFAs, MUFAs, and SFAs were 52.57%, 35.83%, and 11.6% in the SFO and were 46.57%, 38.25%, and 15.18% in the SO, respectively. The concentrations of n-3 and n-6 fatty acids were 0.16% and 52.41% in SFO and 0.26% and 46.31% in SO, respectively. The fatty acid profiles of both types of oils are shown in Table 1.

**Table 1:** Fatty acid composition of sesame and sunflower oils

Variable	Fatty acids	Sesame oil	Sunflower oil
SFAs (%)	Palmitic acid	9.58	6.33
	Stearic acid	4.92	4.25
	Total	15.18	11.6
N-9 MUFAs (%)	Oleic acid	38.25	35.83
	Total	38.25	35.83
N-6 PUFAs (%)	Linoleic acid	46.31	52.41
	Total	46.31	52.41
N-3 PUFAs (%)	Linolenic acid	0.26	0.16
	Total	0.26	0.16
n-6/n-3 PUFAs		178.11	327.56

*SFAs* saturated fatty acids, *MUFAs* monounsaturated fatty acids, *PUFAs* polyunsaturated fatty acids

### Randomization and intervention

After 2 weeks of run-in, 60 individuals were randomly divided into two groups using random allocation method (using permuted block randomization method with block sizes of four) by a third party who did not know about the study and its objectives: (1) 30 individuals in group A received 30 g per day of SO and hypocaloric diet and (2) 30 individuals in group B received 30 g per day of SFO and hypocaloric diet. Then, the intervention was performed for 12 weeks. The types of oils used for the trial and the study objectives were blinded by a person out of this study. The refined, odorless oils were placed in similar, opaque bottles with A and B labels. As a result, participants, facilitators, and researchers became blind to the types of oils being consumed.

The estimated energy requirement (EER) for each individual was calculated, using the Mifflin-St Jeor equation [35], and 500 cal were deducted. The energy distributions consisted of 50–55% carbohydrates, 14–18% protein, and 27–32% fat. Food groups and the food-exchanging were explained to the participants. Half of the oil containers were given to the participants at the beginning of the trial for the first 6 weeks of the study, and the rest were given after the 6-week period. Calibrated cups were given to individuals to consume the exact amount of 30 g of oil per day on their cooked foods or salads for 12 weeks. Four clinical visits were performed at the beginning of run-in and at the beginning, middle, and end of the intervention. Participants were asked not to change their recommended diet, physical activity, and medications during the study. Furthermore, they were asked to report any changes. In addition, participants were followed up by short text messages or phone calls each week. The patients determined to be adhering to the trial were identified by the number of containers they returned and also consumed more than 90% of the provided oils.

### Anthropometric measurements

Body weight was measured using a digital calibrated scale (mode BG 51XXL, Seca, Germany) with light clothes and an accuracy of 0.1 kg. Height was measured in the standing position without shoes while leaning against the wall and shoulders being in normal condition with an accuracy of 0.5 cm using a tape measure mounted on the wall. BMI was calculated based on body weight (kg) divided by height squared (m^2^). Waist circumference (WC) was measured in the middle area between the lowest rib and the upper iliac bone with a non-stretchable measuring tape at the end of a normal exhalation. The hip circumference (HC) is measured as the largest part of the hip, which is also defined as the widest part of the buttocks. The waist to hip ratio (WHR) is calculated by dividing WC over HC. Moreover, index of central obesity (ICO) is calculated by dividing WC over height. To eliminate measurement errors, all measurements are performed by a trained person, three times per visit.

### Dietary intake and physical activity assessment

To assess the diet, participants were instructed in a public session by a nutritionist on how to fill out the food records (the type and amount of all consumed foods and beverages). Participants completed the 3-day weighted food record forms (two weekdays and one weekend day) at the beginning, middle, and end of the intervention to measure dietary nutrients intake, including energy, macronutrients, and micronutrients intake. A total of nine food records for each individual were analyzed using Nutritionist IV software modified for Iranian foods (version 3.5.2, Axxya Systems, Redmond, Washington, DC, USA).

A 3-day self-report record (two weekdays and one weekend day) was used to assess physical activity at the beginning, middle, and end of the intervention. A total of nine physical activity records were obtained for each individual. The participants were asked to maintain their usual physical activity patterns throughout the study. Physical activity data were converted to metabolic equivalents (MET) hour/day, using the updated compendium of physical activities [36].

### Blood pressure measurement

Blood pressure was measured after 10 to 15 min of rest and being away from any excitement and in a sitting position using a mercury sphygmomanometer (model JHSM, China) at the beginning and at the end of the study. To eliminate errors, blood pressure measurements were performed two times per visit by a trained person.

### Laboratory data

At the beginning and the end of the intervention, 10 ml of venous blood from the participants’ left arm was taken after an overnight fast (12 h) between 7 and 10 AM. The blood samples were taken in the sitting position, at the laboratory of Razavi clinic, Shahroud, Iran. It was centrifuged at 3000 rpm for 5 min, and after serum separation, it was kept at − 80 °C until the analysis. TC, TG, HDL-C, and LDL-C were measured using an enzymatic colorimetric method (Pars Azmoon, Tehran, Iran). Non-HDL-C, which is the summation of LDL-C, intermediate-density lipoproteins cholesterol (IDL-C), and very low-density lipoprotein cholesterol (VLDL-C) was determined by the subtraction of HDL-C from TC.

### Statistical analyses

The sample size was calculated for parallel clinical trial studies containing an intervention and a control groups [37]. A sample size of 30 for each group was calculated based on a previous study [38]. It is determined to detect a between-group difference in mean of 0.46 for fatty liver grade as the primary outcome with standard deviation of 0.01 for fatty liver, type one error (*α*) of 0.01, and type two errors (*β*) of 0.20 (power = 80%), and approximately 20% dropout.

Shapiro–Wilk’s test was used to assess the normality of quantitative data distribution. Qualitative and quantitative variables were expressed as frequency report (percentage, %) and mean ± standard deviation, respectively. For the intra-group analyses, paired sample *t*-test (variables with normal distribution) and Wilcoxon test (variables without normal distribution and qualitative variables) were used for comparing the mean of variables. For the inter-group analyses, independent sample *t*-test and Mann–Whitney test were used to examine the mean of quantitative variables with and without normal distribution, respectively. However, chi-square test were used for qualitative variables. Analysis of covariance (ANCOVA and non-parametric ANCOVA) was used to compare the changes of quantitative variables between two groups in the presence of confounders. The potential confounders of age, baseline BMI, physical activity changes, energy intake changes, and baseline values of the variables were included as covariates in the univariate-adjusted model. *p*-value < 0.05 was considered statistically significant. SPSS software (version 25, SPSS Inc., Chicago, IL, USA) was used for data analyses.

## Results

### Study baseline characteristics

**Fig. 1:**
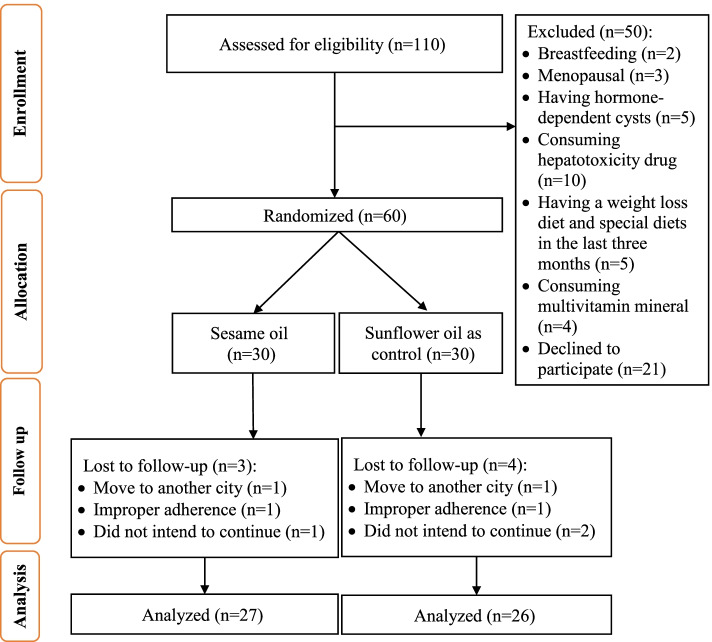
Flow chart of patient recruitment for the clinical trial

Of the total 110 patients with NAFLD assessed for eligibility, 50 patients were excluded based on the exclusion criteria, and 60 participants enrolled, gave their informed written consents, and participated in the trial. They were randomly divided into either the SO group as the intervention group (*n* = 30) or the SFO group as the control group (*n* = 30). Three subjects from the intervention group and four subjects from the control group dropped out during the intervention: adhered improperly (*n* = 2), moved to another city (*n* = 2), and did not intend to continue (*n* = 3). In total, 53 subjects completed this trial and were included in the analysis (Fig. 1). No side effects were observed from oil consumption during the intervention period.

There were no significant differences in age, education level, lipid profile, blood pressure, and anthropometric indices between the two groups at the beginning of the study (Table 2).

**Table 2:** Baseline characteristics of the participants

Quantitative variables		Sesame oil (*n* = 26)	Sunflower oil (*n* = 27)	
		Mean ± Std. deviation	Mean ± Std. deviation	*p*-value**
Age (years)		38.89 ± 6.91	39.35 ± 5.89	NS
Height (cm)		160.96 ± 4.37	161.02 ± 6.19	NS
Body weight (kg)		79.94 ± 9.57	82.91 ± 13.77	NS
BMI (kg/m2)		30.85 ± 3.45	31.86 ± 4.13	NS
WC (cm)		106.39 ± 9.71	108.19 ± 9.93	NS
HC (cm)		112.12 ± 8.32	114.71 ± 10.63	NS
WHR		0.94 ± 0.05	0.94 ± 0.08	NS
ICO		0.66 ± 0.06	0.67 ± 0.06	NS
TG (mg/dL)		145.22 ± 48.65	154.19 ± 44.23	NS
TC (mg/dL)		168.92 ± 26.47	173.53 ± 28.01	NS
LDL-C (mg/dL)		100.92 ± 25.32	106.03 ± 26.35	NS
HDL-C (mg/dL)		54.00 ± 6.07	53.23 ± 2.61	NS
VLDL-C (mg/dL)		29.04 ± 9.73	30.84 ± 8.85	NS
Non-HDL-C (mg/dL)		68.00 ± 8.64	67.50 ± 5.36	NS
TC/HDL-C		3.16 ± 0.63	3.25 ± 0.50	NS
LDL-C/HDL-C		1.91 ± 0.61	1.99 ± 0.48	NS
TG/HDL-C		2.78 ± 1.43	2.91 ± 0.84	NS
SBP (mmHg)		121.22 ± 11.59	126.50 ± 14.02	NS
DBP (mmHg)		77.51 ± 10.37	80.53 ± 10.27	NS
Qualitative variable		*N* (percent)	*N* (percent)	*p*-value**
Education	High school	5 (18.50%)	10 (38.50%)	NS
	Diploma	11 (40.70%)	9 (34.60%)	
	Bachelor	11 (40.70%)	7 (26.90%)	

Between group comparison: *p*-values** were reported based on independent sample *t*-test and Mann–Whitney test for quantitative variables and chi-square test for qualitative variables

*BMI* body mass index, *WC* waist circumference, *HC* hip circumference, *WHR* waist-hip ratio, *ICO* index of central obesity, *TG* triglyceride, *TC* total cholesterol, *LDL-C* low-density lipoprotein cholesterol, *HDL-C* high-density lipoprotein cholesterol, *VLDL-C* very low-density lipoprotein cholesterol, *Non-HDL-C* subtracting HDL-C value from TC, *SBP* systolic blood pressure, *DBP* diastolic blood pressure, *NS* non-significant

### Dietary intake of participants

Table 3 summarizes the physical activity and the intake of macronutrients and micronutrients of participants at the baseline and at the averages of mid- and post-intervention of each group. There was a significant decrease in levels of total energy, carbohydrates, proteins, fat, PUFAs, MUFAs, and SFAs in each group after the intervention. At the end of the intervention, the level of MUFAs in the SO group was significantly higher than the control group (*p* < 0.05), and the level of PUFAs in the control group was significantly higher than the SO (*p* < 0.001). Physical activity remained unchanged throughout the study period in both groups. After the intervention, no significant differences between the two groups were observed for total energy and macronutrient and micronutrient intake except for vitamin E (*p* < 0.05). Vitamin E increased significantly in the SO group compared to the SFO group.

**Table 3:** Physical activity and dietary intake of study participants at baseline and after intervention

		Sesame oil (*n* = 27)	Sunflower oil (*n* = 26)	
Variable	Status	Mean ± Std. deviation	Mean ± Std. deviation	*p*-value**
Energy (Kcal/day)	Before	2166.82 ± 576.94	2258.53 ± 529.81	0.423
	After	1590.83 ± 261.87	1702.95 ± 322.94	0.294
	*p*-value*	< 0.001	< 0.001	
Physical activity (MET hour/day)	Before	33.55 ± 3.65	33.22 ± 3.54	0.734
	After	33.58 ± 3.57	33.42 ± 2.90	0.858
	*p*-value*	0.883	0.562	
Protein (g/day)	Before	78.30 ± 17.31	80.69 ± 25.09	0.687
	After	70.48 ± 13.02	69.63 ± 11.43	0.803
	*p*-value*	0.035	0.028	
Carbohydrates (g/day)	Before	290.75 ± 115.57	307.33 ± 95.64	0.311
	After	207.01 ± 44.40	226.85 ± 50.41	0.160
	*p*-value*	0.001	< 0.001	
Fat (g/day)	Before	81.68 ± 15.97	88.80 ± 16.20	0.070
	After	57.47 ± 7.37	61.55 ± 12.59	0.393
	*p*-value*	< 0.001	< 0.001	
SFAs (g/day)	Before	24.36 ± 8.06	26.10 ± 7.28	0.286
	After	15.25 ± 3.39	15.38 ± 4.45	0.709
	*p*-value*	< 0.001	< 0.001	
MUFAs (g/day)	Before	24.83 ± 4.57	26.19 ± 4.46	0.122
	After	19.65 ± 2.33	18.85 ± 3.49	0.041
	*p*-value*	< 0.001	< 0.001	
PUFAs (g/day)	Before	27.07 ± 5.29	28.99 ± 6.55	0.247
	After	19.81 ± 2.57	23.84 ± 5.87	< 0.001
	*p*-value*	< 0.001	0.001	
Fiber (g/day)	Before	7.74 ± 3.23	8.10 ± 2.93	0.569
	After	6.63 ± 1.98	6.87 ± 1.45	0.615
	*p*-value*	0.077	0.066	
Beta-carotene (μg/d)	Before	987.77 ± 2199.16	1012.47 ± 1208.32	0.233
	After	634.86 ± 857.30	934.50 ± 987.35	0.075
	*p*-value*	0.701	0.657	
Vitamin E (mg/day)	Before	2.63 ± 1.60	3.64 ± 2.25	0.089
	After	11.34 ± 1.14	2.65 ± 1.06	< 0.001
	*p*-value*	< 0.001	0.038	
Vitamin C (mg/day)	Before	85.96 ± 45.06	112.10 ± 113.81	0.817
	After	68.68 ± 35.34	105.34 ± 74.29	0.311
	*p*-value*	0.097	0.909	
Selenium (mg/day)	Before	0.003 ± 0.008	0.003 ± 0.007	0.765
	After	0.002 ± 0.004	0.001 ± 0.003	0.484
	*p*-value*	0.173	0.645	
Zinc (mg/day)	Before	10.25 ± 3.05	10.48 ± 3.49	0.986
	After	8.69 ± 1.85	8.44 ± 1.86	0.466
	*p*-value*	0.019	0.034	

Intragroup analysis: *p*-value* reported based on Paired sample *t*-test and Wilcoxon test for intragroup analysis

Between group comparison: *p*-value** reported based on independent sample *t*-test and Mann–Whitney test

*SFAs* saturated fatty acids, *PUFAs* polyunsaturated fatty acids, *MUFAs* monounsaturated fatty acids, *MET* metabolic equivalent

### The effect of SO on lipid profile, blood pressure, and anthropometric indices

Clinical and anthropometrical variables and their changes after 12 weeks of intervention are presented in Table 4. Given a weight loss diet at the beginning of the study, significant reductions in body weight, BMI, WC, HC, WHR, and ICO were observed in both groups at the end of the study (*p* < 0.001), while no significant differences were observed between the two groups (*p* > 0.05). After 12 weeks of intervention, SBP decreased significantly in both groups, but no significant difference was observed between the two groups (*p* > 0.05). However, changes in DBP (− 4 ± 6.11 mmHg) in the SO group were significant compared to the control group in both unadjusted and adjusted models (*p* < 0.05). At the end of the intervention, HDL-C decreased significantly in both groups (*p* < 0.05), but no significant difference was observed between the groups. No significant changes of LDL-C, TG, TC, VLDL-C, non-HDL-C, LDL-C/HDL-C, and TG/HDL-C were observed at the end of the intervention in both groups (*p* > 0.05). Nevertheless, TC/HDL-C significantly decreased in SO group compared to the control group (*p* < 0.05).

**Table 4:** Analysis of variables after 12 weeks of intervention

Variables	Status	Sesame oil (*n* = 26)	Sunflower oil (*n* = 27)	*p*-value**	*p*-value***
		Mean ± Std. deviation	Mean ± Std. deviation		
Body weight (kg)	After	75.35 ± 9.70	78.94 ± 13.63	0.273	
	Change	− 4.59 ± 2.26	− 3.97 ± 1.79	0.223	0.154
	*p*-value*	< 0.001	< 0.001		
BMI (kg/m2)	After	29.07 ± 3.44	30.32 ± 4.09	0.256	
	Change	− 1.78 ± 0.90	− 1.53 ± 0.69	0.255	0.165
	*p*-value*	< 0.001	< 0.001		
WC (cm)	After	100.48 ± 9.31	103.62 ± 9.73	0.240	
	Change	− 5.91 ± 3.77	− 4.57 ± 2.26	0.135	0.059
	*p*-value*	< 0.001	< 0.001		
HC (cm)	After	109.30 ± 8.11	112.36 ± 10.66	0.244	
	Change	− 2.83 ± 2.06	− 2.35 ± 1.93	0.386	0.352
	*p*-value*	< 0.001	< 0.001		
WHR	After	0.92 ± 0.05	0.93 ± 0.08	0.887	
	Change	− 0.03 ± 0.03	− 0.02 ± 0.01	0.393	0.417
	*p*-value*	< 0.001	< 0.001		
ICO	After	0.62 ± 0.06	0.64 ± 0.06	0.211	
	Change	− 0.04 ± 0.02	− 0.03 ± 0.01	0.126	0.587
	*p*-value*	< 0.001	< 0.001		
TG (mg/dL)	After	154.44 ± 45.24	165.38 ± 56.89	0.470	
	Change	9.22 ± 61.80	11.19 ± 55.67	0.631	0.759
	*p*-value*	0.121	0.216		
TC (mg/dL)	After	162.67 ± 23.14	173.27 ± 26.77	0.132	
	Change	− 6.26 ± 28.52	− 0.27 ± 29.18	0.453	0.100
	*p*-value*	0.274	0.976		
LDL-C (mg/dL)	After	96.41 ± 23.31	107.88 ± 29.33	0.132	
	Change	− 4.52 ± 29.82	1.85 ± 27.93	0.427	0.093
	*p*-value*	0.420	0.810		
HDL-C (mg/dL)	After	51.00 ± 3.11	50.42 ± 4.61	0.842	
	Change	− 3.00 ± 6.73	− 2.81 ± 5.20	0.662	0.992
	*p*-value*	0.023	0.023		
VLDL-C (mg/dL)	After	30.89 ± 9.05	33.08 ± 11.38	0.471	
	Change	1.84 ± 12.36	2.24 ± 11.13	0.631	0.634
	*p*-value*	0.121	0.216		
Non-HDL-C (mg/dL)	After	66.26 ± 7.15	65.38 ± 5.34	0.699	
	Change	− 1.74 ± 10.64	− 2.12 ± 7.74	0.838	0.415
	*p*-value*	0.286	0.094		
TC/HDL-C	After	3.06 ± 0.55	3.50 ± 0.92	0.056	
	Change	− 0.11 ± 0.83	0.25 ± 0.85	0.236	0.039
	*p*-value*	0.716	0.152		
LDL-C/HDL-C	After	1.90 ± 0.50	2.31 ± 0.92	0.066	
	Change	− 0.01 ± 0.75	0.32 ± 0.75	0.335	0.067
	*p*-value*	0.934	0.056		
TG/HDL-C	After	3.08 ± 1.10	3.42 ± 1.73	0.493	
	Change	0.30 ± 1.79	0.51 ± 1.61	0.735	0.658
	*p*-value*	0.072	0.091		
SBP (mmHg)	After	114.44 ± 12.52	119.92 ± 12.28	0.114	
	Change	− 6.78 ± 10.23	− 6.58 ± 12.08	0.948	0.459
	*p*-value*	0.002	0.010		
DBP (mmHg)	After	73.52 ± 10.54	80.27 ± 7.94	0.011	
	Change	− 4.00 ± 6.11	− 0.27 ± 10.17	0.110	0.031
	*p*-value*	0.002	0.890		

Changes imply for after minus before. Intragroup analysis: *p*-values* were reported based on Paired sample *t*-test and Wilcoxon test

Between group comparison for crude model: *p*-values** were reported based on Mann–Whitney test for quantitative variables and chi-square test for qualitative variables

Between group comparison for adjusted model (age, baseline BMI, physical activity changes, energy intake changes, and baseline values of the variable): *p*-values*** were reported based on non-parametric ANCOVA for quantitative variables

*BMI* body mass index, *WC* waist circumference, *HC* hip circumference, *WHR* waist-hip ratio, *ICO* index of central obesity, *TG* triglyceride, *TC* total cholesterol, *LDL-C* low-density lipoprotein cholesterol, *HDL-C* high-density lipoprotein cholesterol, *VLDL-C* very low-density lipoprotein cholesterol, *Non-HDL-C* subtracting HDL-C value from TC, *SBP* systolic blood pressure, *DBP* diastolic blood pressure

## Discussion

The purpose of this randomized controlled trial was to compare the effects of SO in the context of a weight loss program on lipid profile, blood pressure, and anthropometric indices in women with NAFLD. Following 12 weeks of SO consumption, despite having no effect on lipid profile, favorable changes in SBP, DBP, body weight, WC, HC, WHR, and ICO were observed. Similar results were observed following SFO intake, except for DBP with no changes. At the end of the intervention, HDL-C decreased in both groups. Comparisons between treatment groups showed significant reductions in DBP and TC/HDL-C in SO group compared to SFO group.

### Lipid profile

In our study, TC and LDL-C levels decreased in the SO group but they were not statistically significant. However, the ratio of TC to HDL-C was significantly reduced in the SO group compared to the control group. In support of our results, in a parallel study, Khajehdehi et al. examined patients with kidney problems that ingested SO for 8 weeks. They showed TC, TG, and LDL-C levels remained unchanged at end of the intervention [29]. On the contrary, a number of studies with parallel design in patients with metabolic disorders that consumed SO showed significant decreases in TC, LDL-C, and TG levels compared to the control group [20, 23, 26, 28]. Differences in participants’ diseases, dosages of SO, and the particular study designs may have been the reasons for the differences between the results of these studies and our study. Perhaps another reason for the lack of significant reductions in TC and LDL-C is their normal levels in the base line that may have not allowed seeing the best benefit of SO consumption. While, two meta-analysis studies showed the beneficial effect of sesame on lipid profile [39, 40].

There were some probable mechanisms, which can show the hypocholesterolemic effects of SO. SO is rich in MUFA [20] and various studies have shown that high levels MUFA inhibits lipogenesis by increasing the oxidation of fatty acids by activating the peroxisome proliferator-activated receptor alpha or by decreasing the activation of the sterol regulatory element binding protein [19]. Sankar’s et al. stated that the decrease in LDL-C and TC may be due to high levels of MUFA in SO [28]. SO lignans belong to phytochemical family [41]. A human study showed that sesamin, a lignan in SO, significantly reduced LDL-C [42]. In this study, it was suggested that sesamin can potentially reduce 3-hydroxy-3-methyl-glutaryl-coenzyme A reductase activity [42]. In our study, dietary assessment showed the level of MUFAs in the SO group was significantly higher than the control group at the end of the intervention. However, we did not examine the fatty acid content of red blood cells (RBC), which is one of the limitations of our study.

Studies showed that γ-tocopherol in SO can cause platelet aggregation reduction, LDL-C oxidation, intra-arterial thrombosis delay [43], and cholesterol biosynthesis inhibition [44]. Our study showed that vitamin E intake was increased with SO consumption. However, we did not examine serum levels of vitamin E, which is another limitation of our study.

In our study, HDL-C level significantly reduced in both groups but these reductions were not much different between the two groups. A number of studies have shown that consuming SO increases HDL-C levels [25–27], while other studies have shown that it remains unchanged [20, 29]. The reason for HDL-C level reduction in our study may be due to the adherence to low-calorie diets and the reduction in fat intake [45].

### Blood pressure

In this trial, SBP decreased significantly in both groups and DBP decreased significantly in the SO group compared to the control group. Some studies have shown beneficial effects of SO consumption on blood pressure [20, 22, 23, 28], while one study has not shown this effect [29]. In our study, the significant reduction in DBP in SO group compared to the control group may be due to the oil composition.

A systematic review done by Cardoso et al. reported positive effects of dietary intake of sesame derivatives on blood pressure [46]. Multiple possible mechanisms explain the beneficial effects of SO on blood pressure. The antihypertensive effect of SO is related to antioxidant lignans, including sesamolin, sesamol, episesamin, and sesamin as well as its multiple tocopherol homologs [α-tocopherol, δ-tocopherol, γ-tocopherol, and tocotrienols] content that break the radical chain in membranes and lipoproteins [20, 28]. A study showed that black sesame meal capsule supplementations for 4 weeks in prehypertension patients leads to an increase in serum levels of vitamin E and a decrease in blood pressure [47]. Experimental models showed that the antihypertensive effects of sesamin were related to its antagonistic activity on calcium channels [48–50]. Another experimental study showed that sesamin increases the concentration of nitric oxide and inhibits the production of endothelin-1 by endothelial cells [50]. Vitamin E in SO improves endothelial dysfunction by reducing free radicals [51] and leads to an increase in vasodilator or nitric oxide factors and consequently reduces blood pressure [52].

### Anthropometric indices

This trial showed significant decreases in body weight, BMI, HC, WC, WHR, and ICO in both groups. However, no significant differences in these indicators were observed between the two groups. In support of our results, in a well-designed study conducting by Raeisi et al., which assessed the effect of SO consumption for 9 weeks in adults, no change in body weight and BMI were observed [53]. Also, Yazdi et al. showed similar results [54]. On the contrary, Sankar et al. showed that the consumption of 35 g SO for 45 days in hypertension patients has beneficial effects on body weight and BMI [28]. It should be considered that this study had no control group and also the participants and researchers were not blind to the oils. In a parallel study, Sankar et al. examined the effect of 35 g SO consumption for 60 days on hypertension patients and observed that body weight and BMI decreased after the intervention [23]. In this study, the results may have been affected due to the concomitant use of nifedipine with SO. A meta-analysis that evaluated the effects of sesame seed and its products on BMI and body weight showed that SO reduced body weight and BMI. The weak design and the small number of studies were the two limitations of this meta-analysis, which may make it results become unreliable.

### Strengths and limitations

Our study has several strengths: (1) the methodology and design of previous studies were not rigorous due to the lack of allocation concealment, blinding of participants and personnel, physical activity, and dietary intake assessments. We did all of them to minimize the potential risk of biases. (2) All participants were female and aged 20–50 years old who were almost homogeneous in physiological and hormonal conditions.

Our study has several limitations that should be taken into account when interpreting the results: (1) we did not examine the fatty acid content of RBC and serum levels of vitamin E. Therefore, future research is needed to use more objective methods of assessing the type of fatty acids (MUFAs and PUFAs) in RBC and serum levels of vitamin E to confirm adherence [55, 56]. (2) We did not include oils with high saturated fatty acids such as hydrogenated oils and palm oil found in the western diet. It does not seem ethical to use unhealthy oils for a long time (12 weeks) in clinical trials. (3) Normal levels of blood lipids of the patients may have not allowed seeing the best benefit from the administered oil.

## Conclusions

The present investigation provides evidences that SO may be effective in improvement of DBP and TC/HDL-C, whereas no significant effects were observed in anthropometric indices, SBP, TG, TC, LDL-C, HDL-C, VLDL-C, non-HDL-C, LDL-C/HDL-C, and TG/HDL-C compared to the control group. Further well-designed studies should be conducted to confirm these results. Also, it is suggested that future studies examine the effects of SO on fatty liver diseases undergoing an isocaloric diet.

## Data Availability

Available upon request.

## Abbreviations

NAFLD: Non-alcoholic fatty liver
SO: Sesame oil
SFO: Sunflower oil
SBP: Systolic blood pressure
DBP: Systolic blood pressure
TC: Total cholesterol
HDL-C: High-density lipoprotein cholesterol
TG: Triglyceride
LDL-C: Low-density lipoprotein cholesterol
IRCT: Iranian registry of clinical trials
CVD: Cardiovascular disease
MUFAs: Monounsaturated fatty acids
PUFAs: Polyunsaturated fatty acids
SPIRIT: Standard Protocol Items: Recommendations for Interventional Trials
EER: Estimated energy requirement
WC: Waist circumference
HC: Hip circumference
WHR: Waist to hip ratio
ICO: Index of central obesity
MET: Metabolic equivalents
IDL-C: Intermediate-density lipoproteins cholesterol
VLDL-C: Very low-density lipoprotein cholesterol
ANCOVA: Analysis of covariance
RBC: Red blood cells
